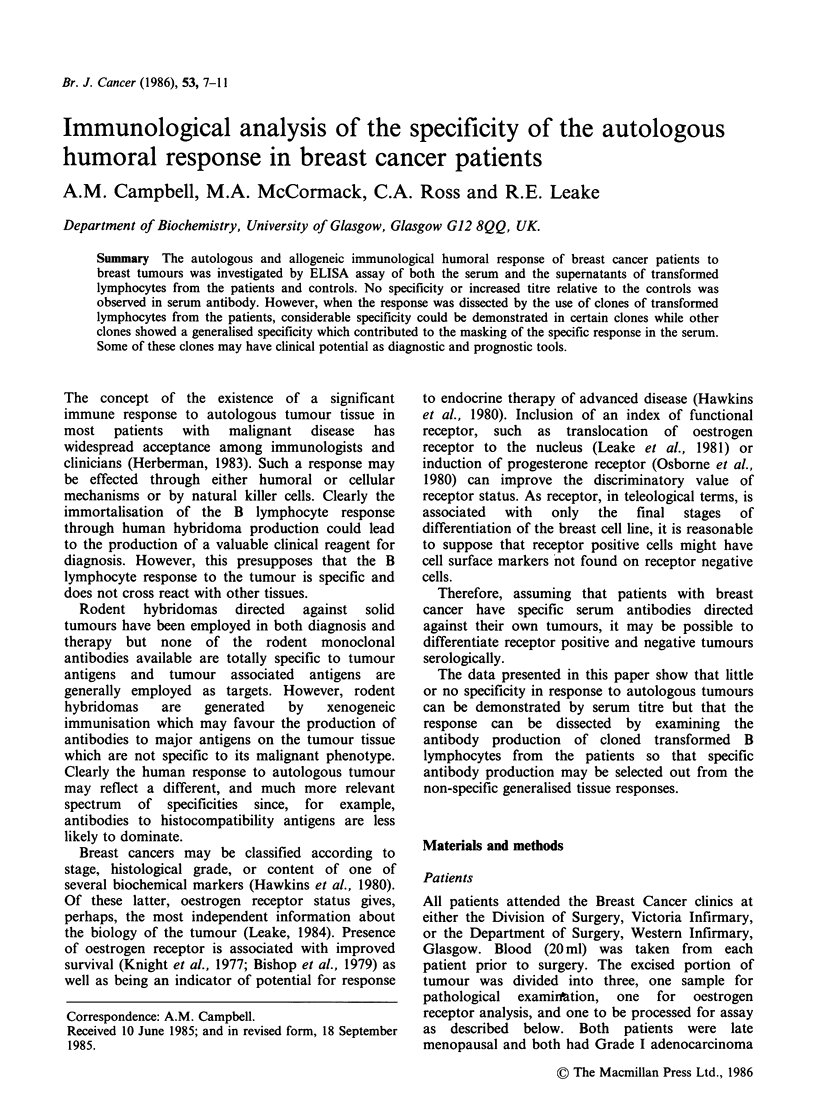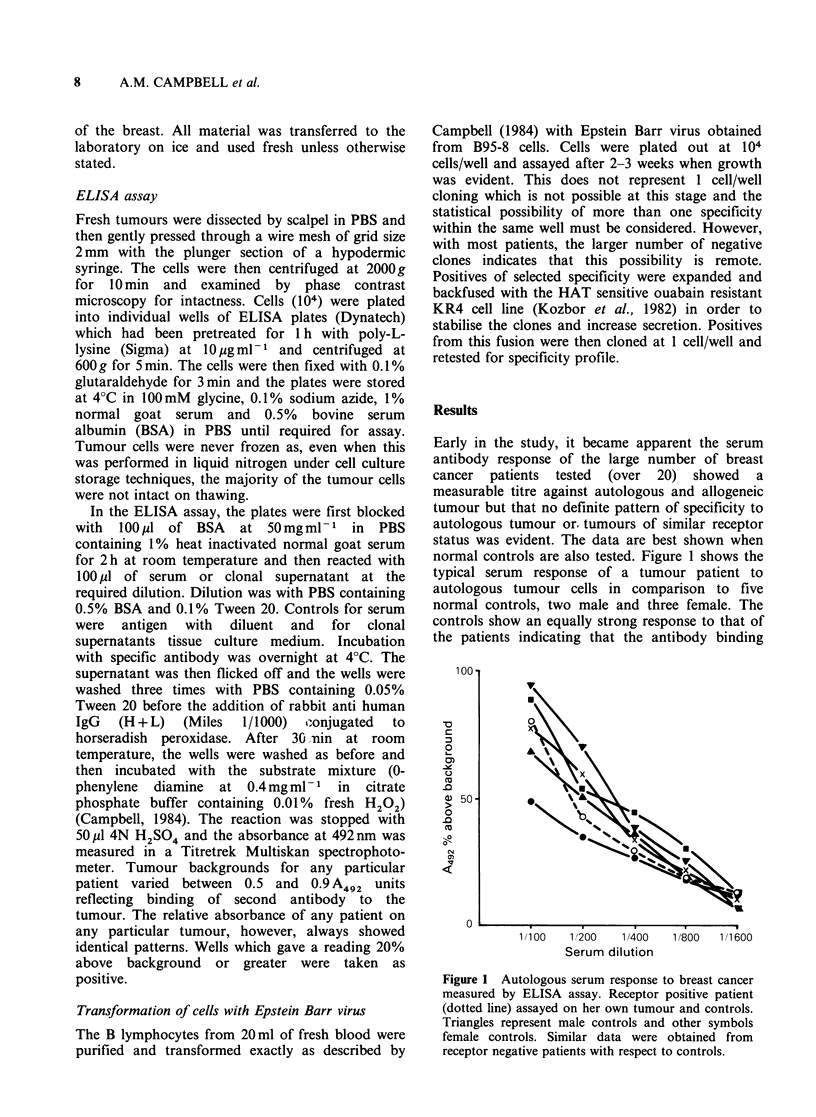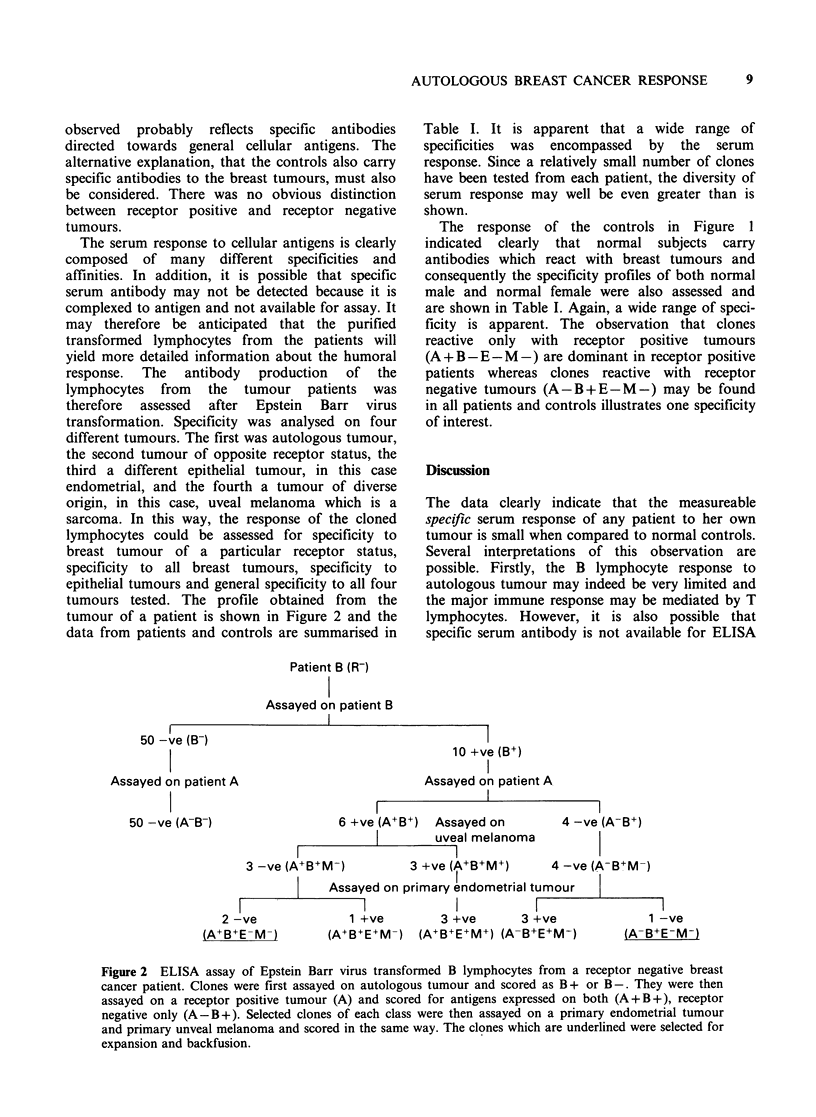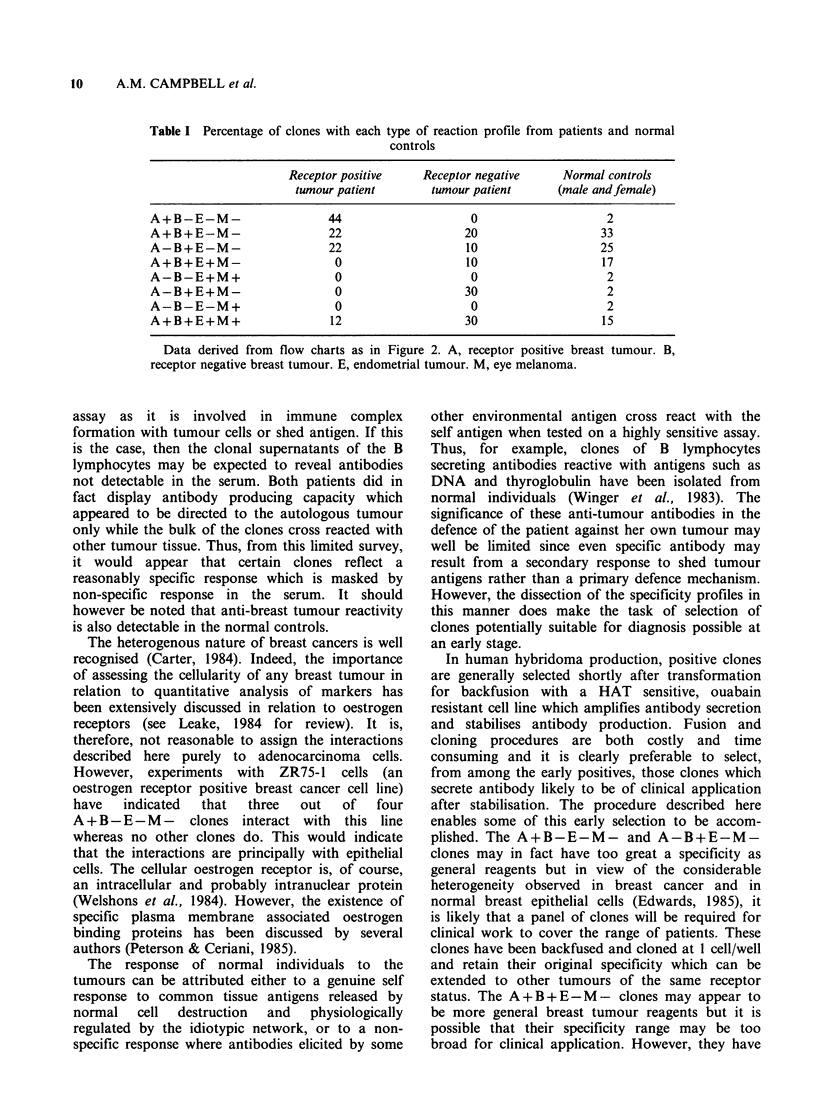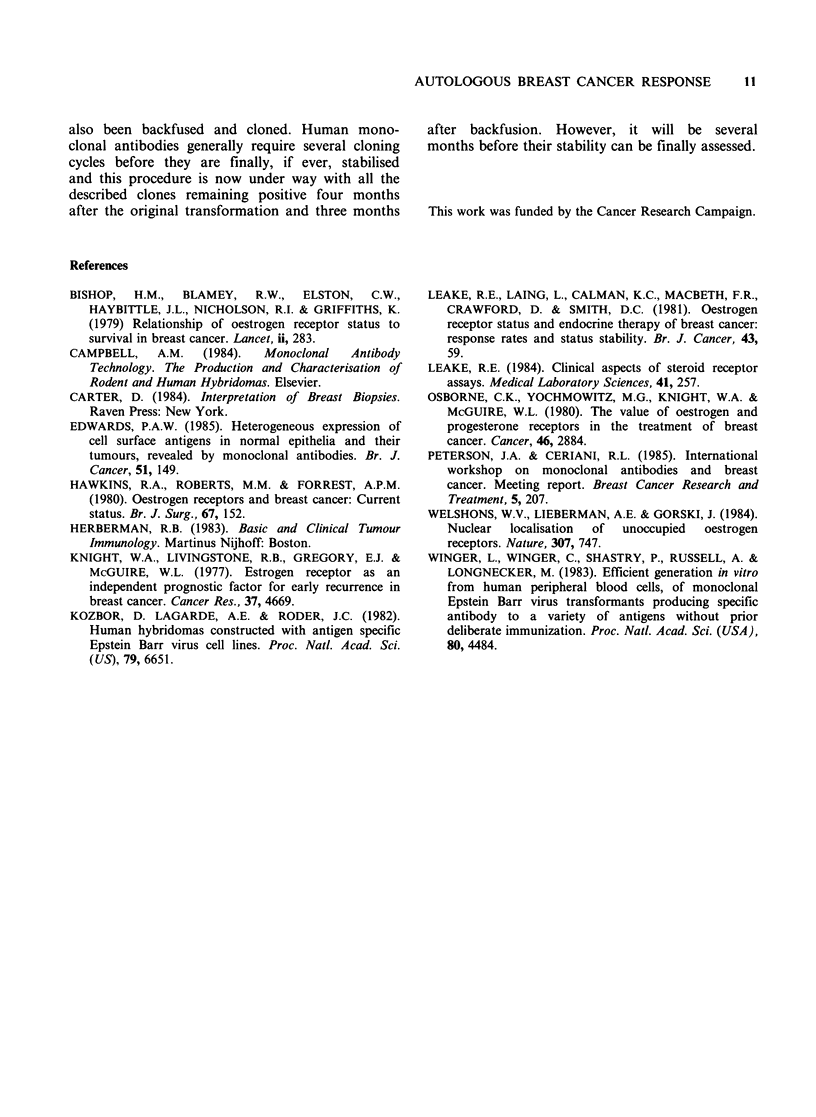# Immunological analysis of the specificity of the autologous humoral response in breast cancer patients.

**DOI:** 10.1038/bjc.1986.2

**Published:** 1986-01

**Authors:** A. M. Campbell, M. A. McCormack, C. A. Ross, R. E. Leake

## Abstract

The autologous and allogeneic immunological humoral response of breast cancer patients to breast tumours was investigated by ELISA assay of both the serum and the supernatants of transformed lymphocytes from the patients and controls. No specificity or increased titre relative to the controls was observed in serum antibody. However, when the response was dissected by the use of clones of transformed lymphocytes from the patients, considerable specificity could be demonstrated in certain clones while other clones showed a generalised specificity which contributed to the masking of the specific response in the serum. Some of these clones may have clinical potential as diagnostic and prognostic tools.


*Br. J. Cancer* (1986), **53**, 7–11

### Immunological analysis of the specificity of the autologous humoral response in breast cancer patients

A.M. Campbell, M.A. McCormack, C.A. Ross and R.E. Leake

*Department of Biochemistry, University of Glasgow, Glasgow G12 8QQ, UK.*

**Summary** The autologous and allogeneic immunological humoral response of breast cancer patients to breast tumours was investigated by ELISA assay of both the serum and the supernatants of transformed lymphocytes from the patients and controls. No specificity or increased titre relative to the controls was observed in serum antibody. However, when the response was dissected by the use of clones of transformed lymphocytes from the patients, considerable specificity could be demonstrated in certain clones while other clones showed a generalised specificity which contributed to the masking of the specific response in the serum. Some of these clones may have clinical potential as diagnostic and prognostic tools.

The concept of the existence of a significant immune response to autologous tumour tissue in most patients with malignant disease has widespread acceptance among immunologists and clinicians (Herberman, 1983). Such a response may be effected through either humoral or cellular mechanisms or by natural killer cells. Clearly the immortalisation of the B lymphocyte response through human hybridoma production could lead to the production of a valuable clinical reagent for diagnosis. However, this presupposes that the B lymphocyte response to the tumour is specific and does not cross react with other tissues.

Rodent hybridomas directed against solid tumours have been employed in both diagnosis and therapy but none of the rodent monoclonal antibodies available are totally specific to tumour antigens and tumour associated antigens are generally employed as targets. However, rodent hybridomas are generated by xenogeneic immunisation which may favour the production of antibodies to major antigens on the tumour tissue which are not specific to its malignant phenotype. Clearly the human response to autologous tumour may reflect a different, and much more relevant spectrum of specificities since, for example, antibodies to histocompatibility antigens are less likely to dominate.

Breast cancers may be classified according to stage, histological grade, or content of one of several biochemical markers (Hawkins *et al.*, 1980). Of these latter, oestrogen receptor status gives, perhaps, the most independent information about the biology of the tumour (Leake, 1984). Presence of oestrogen receptor is associated with improved survival (Knight *et al.*, 1977; Bishop *et al.*, 1979) as well as being an indicator of potential for response to endocrine therapy of advanced disease (Hawkins *et al.*, 1980). Inclusion of an index of functional receptor, such as translocation of oestrogen receptor to the nucleus (Leake *et al.*, 1981) or induction of progesterone receptor (Osborne *et al.*, 1980) can improve the discriminatory value of receptor status. As receptor, in teleological terms, is associated with only the final stages of differentiation of the breast cell line, it is reasonable to suppose that receptor positive cells might have cell surface markers not found on receptor negative cells.

Therefore, assuming that patients with breast cancer have specific serum antibodies directed against their own tumours, it may be possible to differentiate receptor positive and negative tumours serologically.

The data presented in this paper show that little or no specificity in response to autologous tumours can be demonstrated by serum titre but that the response can be dissected by examining the antibody production of cloned transformed B lymphocytes from the patients so that specific antibody production may be selected out from the non-specific generalised tissue responses.

### Materials and methods

### *Patients*

All patients attended the Breast Cancer clinics at either the Division of Surgery, Victoria Infirmary, or the Department of Surgery, Western Infirmary, Glasgow. Blood (20 ml) was taken from each patient prior to surgery. The excised portion of tumour was divided into three, one sample for pathological examination, one for oestrogen receptor analysis, and one to be processed for assay as described below. Both patients were late menopausal and both had Grade I adenocarcinoma

Correspondence: A.M. Campbell.
Received 10 June 1985; and in revised form, 18 September 1985.

© The Macmillan Press Ltd., 1986

of the breast. All material was transferred to the laboratory on ice and used fresh unless otherwise stated.

*ELISA assay*

Fresh tumours were dissected by scalpel in PBS and then gently pressed through a wire mesh of grid size 2mm with the plunger section of a hypodermic syringe. The cells were then centrifuged at 2000 *g* for 10min and examined by phase contrast microscopy for intactness. Cells ($10^4$) were plated into individual wells of ELISA plates (Dynatech) which had been pretreated for 1 h with poly-L-lysine (Sigma) at $10 \mu g ml^{-1}$ and centrifuged at 600 *g* for 5 min. The cells were then fixed with 0.1% glutaraldehyde for 3 min and the plates were stored at 4°C in 100mM glycine, 0.1% sodium azide, 1% normal goat serum and 0.5% bovine serum albumin (BSA) in PBS until required for assay. Tumour cells were never frozen as, even when this was performed in liquid nitrogen under cell culture storage techniques, the majority of the tumour cells were not intact on thawing.

In the ELISA assay, the plates were first blocked with 100 µl of BSA at $50 mg ml^{-1}$ in PBS containing 1% heat inactivated normal goat serum for 2 h at room temperature and then reacted with 100 µl of serum or clonal supernatant at the required dilution. Dilution was with PBS containing 0.5% BSA and 0.1% Tween 20. Controls for serum were antigen with diluent and for clonal supernatants tissue culture medium. Incubation with specific antibody was overnight at 4°C. The supernatant was then flicked off and the wells were washed three times with PBS containing 0.05% Tween 20 before the addition of rabbit anti human IgG (H+L) (Miles 1/1000) conjugated to horseradish peroxidase. After 30 min at room temperature, the wells were washed as before and then incubated with the substrate mixture (0-phenylene diamine at $0.4 mg ml^{-1}$ in citrate phosphate buffer containing 0.01% fresh $H_2O_2$) (Campbell, 1984). The reaction was stopped with 50 µl 4N $H_2SO_4$ and the absorbance at 492 nm was measured in a Titretrek Multiskan spectrophotometer. Tumour backgrounds for any particular patient varied between 0.5 and 0.9 $A_{492}$ units reflecting binding of second antibody to the tumour. The relative absorbance of any patient on any particular tumour, however, always showed identical patterns. Wells which gave a reading 20% above background or greater were taken as positive.

*Transformation of cells with Epstein Barr virus*

The B lymphocytes from 20 ml of fresh blood were purified and transformed exactly as described by Campbell (1984) with Epstein Barr virus obtained from B95-8 cells. Cells were plated out at $10^4$ cells/well and assayed after 2–3 weeks when growth was evident. This does not represent 1 cell/well cloning which is not possible at this stage and the statistical possibility of more than one specificity within the same well must be considered. However, with most patients, the larger number of negative clones indicates that this possibility is remote. Positives of selected specificity were expanded and backfused with the HAT sensitive ouabain resistant KR4 cell line (Kozbor *et al.*, 1982) in order to stabilise the clones and increase secretion. Positives from this fusion were then cloned at 1 cell/well and retested for specificity profile.

### Results

Early in the study, it became apparent the serum antibody response of the large number of breast cancer patients tested (over 20) showed a measurable titre against autologous and allogeneic tumour but that no definite pattern of specificity to autologous tumour or tumours of similar receptor status was evident. The data are best shown when normal controls are also tested. Figure 1 shows the typical serum response of a tumour patient to autologous tumour cells in comparison to five normal controls, two male and three female. The controls show an equally strong response to that of the patients indicating that the antibody binding

**Figure 1** Autologous serum response to breast cancer measured by ELISA assay. Receptor positive patient (dotted line) assayed on her own tumour and controls. Triangles represent male controls and other symbols female controls. Similar data were obtained from receptor negative patients with respect to controls.

observed probably reflects specific antibodies directed towards general cellular antigens. The alternative explanation, that the controls also carry specific antibodies to the breast tumours, must also be considered. There was no obvious distinction between receptor positive and receptor negative tumours.

The serum response to cellular antigens is clearly composed of many different specificities and affinities. In addition, it is possible that specific serum antibody may not be detected because it is complexed to antigen and not available for assay. It may therefore be anticipated that the purified transformed lymphocytes from the patients will yield more detailed information about the humoral response. The antibody production of the lymphocytes from the tumour patients was therefore assessed after Epstein Barr virus transformation. Specificity was analysed on four different tumours. The first was autologous tumour, the second tumour of opposite receptor status, the third a different epithelial tumour, in this case endometrial, and the fourth a tumour of diverse origin, in this case, uveal melanoma which is a sarcoma. In this way, the response of the cloned lymphocytes could be assessed for specificity to breast tumour of a particular receptor status, specificity to all breast tumours, specificity to epithelial tumours and general specificity to all four tumours tested. The profile obtained from the tumour of a patient is shown in Figure 2 and the data from patients and controls are summarised in Table I. It is apparent that a wide range of specificities was encompassed by the serum response. Since a relatively small number of clones have been tested from each patient, the diversity of serum response may well be even greater than is shown.

The response of the controls in Figure 1 indicated clearly that normal subjects carry antibodies which react with breast tumours and consequently the specificity profiles of both normal male and normal female were also assessed and are shown in Table I. Again, a wide range of specificity is apparent. The observation that clones reactive only with receptor positive tumours (A+B−E−M−) are dominant in receptor positive patients whereas clones reactive with receptor negative tumours (A−B+E−M−) may be found in all patients and controls illustrates one specificity of interest.

### Discussion

The data clearly indicate that the measureable *specific* serum response of any patient to her own tumour is small when compared to normal controls. Several interpretations of this observation are possible. Firstly, the B lymphocyte response to autologous tumour may indeed be very limited and the major immune response may be mediated by T lymphocytes. However, it is also possible that specific serum antibody is not available for ELISA

**Figure 2** ELISA assay of Epstein Barr virus transformed B lymphocytes from a receptor negative breast cancer patient. Clones were first assayed on autologous tumour and scored as B+ or B−. They were then assayed on a receptor positive tumour (A) and scored for antigens expressed on both (A+B+), receptor negative only (A−B+). Selected clones of each class were then assayed on a primary endometrial tumour and primary unveal melanoma and scored in the same way. The clones which are underlined were selected for expansion and backfusion.

**Table I** Percentage of clones with each type of reaction profile from patients and normal controls

| | *Receptor positive tumour patient* | *Receptor negative tumour patient* | *Normal controls (male and female)* |
|---|---|---|---|
| A+B−E−M− | 44 | 0 | 2 |
| A+B+E−M− | 22 | 20 | 33 |
| A−B+E−M− | 22 | 10 | 25 |
| A+B+E+M− | 0 | 10 | 17 |
| A−B−E+M+ | 0 | 0 | 2 |
| A−B+E+M− | 0 | 30 | 2 |
| A−B−E−M+ | 0 | 0 | 2 |
| A+B+E+M+ | 12 | 30 | 15 |

Data derived from flow charts as in Figure 2. A, receptor positive breast tumour. B, receptor negative breast tumour. E, endometrial tumour. M, eye melanoma.

assay as it is involved in immune complex formation with tumour cells or shed antigen. If this is the case, then the clonal supernatants of the B lymphocytes may be expected to reveal antibodies not detectable in the serum. Both patients did in fact display antibody producing capacity which appeared to be directed to the autologous tumour only while the bulk of the clones cross reacted with other tumour tissue. Thus, from this limited survey, it would appear that certain clones reflect a reasonably specific response which is masked by non-specific response in the serum. It should however be noted that anti-breast tumour reactivity is also detectable in the normal controls.

The heterogenous nature of breast cancers is well recognised (Carter, 1984). Indeed, the importance of assessing the cellularity of any breast tumour in relation to quantitative analysis of markers has been extensively discussed in relation to oestrogen receptors (see Leake, 1984 for review). It is, therefore, not reasonable to assign the interactions described here purely to adenocarcinoma cells. However, experiments with ZR75-1 cells (an oestrogen receptor positive breast cancer cell line) have indicated that three out of four A+B−E−M− clones interact with this line whereas no other clones do. This would indicate that the interactions are principally with epithelial cells. The cellular oestrogen receptor is, of course, an intracellular and probably intranuclear protein (Welshons *et al.*, 1984). However, the existence of specific plasma membrane associated oestrogen binding proteins has been discussed by several authors (Peterson & Ceriani, 1985).

The response of normal individuals to the tumours can be attributed either to a genuine self response to common tissue antigens released by normal cell destruction and physiologically regulated by the idiotypic network, or to a non-specific response where antibodies elicited by some other environmental antigen cross react with the self antigen when tested on a highly sensitive assay. Thus, for example, clones of B lymphocytes secreting antibodies reactive with antigens such as DNA and thyroglobulin have been isolated from normal individuals (Winger *et al.*, 1983). The significance of these anti-tumour antibodies in the defence of the patient against her own tumour may well be limited since even specific antibody may result from a secondary response to shed tumour antigens rather than a primary defence mechanism. However, the dissection of the specificity profiles in this manner does make the task of selection of clones potentially suitable for diagnosis possible at an early stage.

In human hybridoma production, positive clones are generally selected shortly after transformation for backfusion with a HAT sensitive, ouabain resistant cell line which amplifies antibody secretion and stabilises antibody production. Fusion and cloning procedures are both costly and time consuming and it is clearly preferable to select, from among the early positives, those clones which secrete antibody likely to be of clinical application after stabilisation. The procedure described here enables some of this early selection to be accomplished. The A+B−E−M− and A−B+E−M− clones may in fact have too great a specificity as general reagents but in view of the considerable heterogeneity observed in breast cancer and in normal breast epithelial cells (Edwards, 1985), it is likely that a panel of clones will be required for clinical work to cover the range of patients. These clones have been backfused and cloned at 1 cell/well and retain their original specificity which can be extended to other tumours of the same receptor status. The A+B+E−M− clones may appear to be more general breast tumour reagents but it is possible that their specificity range may be too broad for clinical application. However, they have also been backfused and cloned. Human monoclonal antibodies generally require several cloning cycles before they are finally, if ever, stabilised and this procedure is now under way with all the described clones remaining positive four months after the original transformation and three months after backfusion. However, it will be several months before their stability can be finally assessed.

This work was funded by the Cancer Research Campaign.

### References

BISHOP, H.M., BLAMEY, R.W., ELSTON, C.W., HAYBITTLE, J.L., NICHOLSON, R.I. & GRIFFITHS, K. (1979) Relationship of oestrogen receptor status to survival in breast cancer. *Lancet*, **ii**, 283.

CAMPBELL, A.M. (1984). *Monoclonal Antibody Technology. The Production and Characterisation of Rodent and Human Hybridomas*. Elsevier.

CARTER, D. (1984). *Interpretation of Breast Biopsies*. Raven Press: New York.

EDWARDS, P.A.W. (1985). Heterogeneous expression of cell surface antigens in normal epithelia and their tumours, revealed by monoclonal antibodies. *Br. J. Cancer*, **51**, 149.

HAWKINS, R.A., ROBERTS, M.M. & FORREST, A.P.M. (1980). Oestrogen receptors and breast cancer: Current status. *Br. J. Surg.*, **67**, 152.

HERBERMAN, R.B. (1983). *Basic and Clinical Tumour Immunology*. Martinus Nijhoff: Boston.

KNIGHT, W.A., LIVINGSTONE, R.B., GREGORY, E.J. & McGUIRE, W.L. (1977). Estrogen receptor as an independent prognostic factor for early recurrence in breast cancer. *Cancer Res.*, **37**, 4669.

KOZBOR, D. LAGARDE, A.E. & RODER, J.C. (1982). Human hybridomas constructed with antigen specific Epstein Barr virus cell lines. *Proc. Natl. Acad. Sci.* (*US*), **79**, 6651.

LEAKE, R.E., LAING, L., CALMAN, K.C., MACBETH, F.R., CRAWFORD, D. & SMITH, D.C. (1981). Oestrogen receptor status and endocrine therapy of breast cancer: response rates and status stability. *Br. J. Cancer*, **43**, 59.

LEAKE, R.E. (1984). Clinical aspects of steroid receptor assays. *Medical Laboratory Sciences*, **41**, 257.

OSBORNE, C.K., YOCHMOWITZ, M.G., KNIGHT, W.A. & McGUIRE, W.L. (1980). The value of oestrogen and progesterone receptors in the treatment of breast cancer. *Cancer*, **46**, 2884.

PETERSON, J.A. & CERIANI, R.L. (1985). International workshop on monoclonal antibodies and breast cancer. Meeting report. *Breast Cancer Research and Treatment*, **5**, 207.

WELSHONS, W.V., LIEBERMAN, A.E. & GORSKI, J. (1984). Nuclear localisation of unoccupied oestrogen receptors. *Nature*, **307**, 747.

WINGER, L., WINGER, C., SHASTRY, P., RUSSELL, A. & LONGNECKER, M. (1983). Efficient generation *in vitro* from human peripheral blood cells, of monoclonal Epstein Barr virus transformants producing specific antibody to a variety of antigens without prior deliberate immunization. *Proc. Natl. Acad. Sci.* (*USA*), **80**, 4484.